# Modeling the Effects of Integrating Larval Habitat Source Reduction and Insecticide Treated Nets for Malaria Control

**DOI:** 10.1371/journal.pone.0006921

**Published:** 2009-09-09

**Authors:** Laith Yakob, Guiyun Yan

**Affiliations:** Program in Public Health, University of California Irvine, Irvine, California, United States of America; Université Pierre et Marie Curie, France

## Abstract

Integrated vector management for malaria control has received a lot of recent interest. Attacking multiple points in the transmission cycle is hoped to act synergistically and improve upon current single-tool interventions based on the use of insecticide-treated bed nets (ITNs). In the present study, we theoretically examined the application of larval habitat source reduction with ITNs in reducing malaria transmission. We selected this type of environmental management to complement ITNs because of a potential secondary mode of action that both control strategies share. In addition to increasing vector mortality, ITNs reduce the rate at which female mosquitoes locate human hosts for blood feeding, thereby extending their gonotrophic cycle. Similarly, while reducing adult vector emergence and abundance, source reduction of larval habitats may prolong the cycle duration by extending delays in locating oviposition sites. We found, however, that source reduction of larval habitats only operates through this secondary mode of action when habitat density is below a critical threshold. Hence, we illustrate how this strategy becomes increasingly effective when larval habitats are limited. We also demonstrate that habitat source reduction is better suited to human populations of higher density and in the presence of insecticide resistance or when the insecticidal properties of ITNs are depleted.

## Introduction

Vector management is the primary means of malaria prevention and control in Africa [1], and has become increasingly important following the emergence and rapid spread of drug resistant and multi-drug resistant parasites [2]. Pyrethrum insecticide-treated nets (ITNs), particularly the long-lasting insecticidal nets (LLIN), is the preferred tool for reducing malaria transmission and alleviating disease burden [3], [4]. In addition to providing a physical barrier to reduce human-mosquito contact, the nets increase vector mortality, meaning fewer mosquitoes survive the duration necessary to become infectious. High coverage of ITNs thereby benefits the whole local community, not just the users, as demonstrated in a number of studies in Africa [5]–[7].

There are, however, limitations to ITNs as a stand-alone strategy for malaria control. Insecticidal properties of ITNs have been shown to diminish considerably after just 6 months of use [8], and rates of re-treatment with insecticide and community compliance are often discouraging [9]. Further, the emergence and spread of resistance to pyrethroid insecticides in anopheline mosquitoes is of major concern to the long-term effectiveness of ITNs [10]. Recently, emphasis has been put on using multiple mosquito control strategies in unison to reduce dependence on this single, insecticide-based tool [1]. Attacking the transmission cycle at multiple points is expected to act synergistically to enhance current malaria programs, and has rekindled hopes for malaria elimination [11]. In order to accelerate the uptake of integrated vector management (IVM), theoretical studies are required to analyze the efficacy of potential combinations of various tools before the more costly and time-consuming empirical evaluations can take place. Here, we describe a simple mathematical model that examines the effects on malaria transmission of combining source reduction of mosquito larval habitats with ITNs. We choose larval habitat management to complement ITN programs because of the recent attention habitat source reduction has received [12]–[16]. This strategy involves the use of ecological means to reduce the number of productive larval habitats, thereby decreasing the emergence rate of adult vectors. Historically, environmental management has had great success in eradicating malaria from the US and Europe [11], [17], and in some local elimination programs in Africa [18], [19].

Larval habitat source reduction is expected to impact the transmission of malaria on multiple fronts. In addition to reducing the growth potential of the mosquito population, larval habitat source management will reduce the rate at which gravid females will encounter oviposition sites. This is an important point first raised by Killeen et al. (2004) who accounted for this additional delay in their model by reducing the average daily human bite rate of mosquitoes. In their study, multiple vector management scenarios were assessed including water management, larvicide application, physical domestic protection and zooprophylaxis, and it was concluded that all measures in combination are 100 fold more effective in reducing malaria transmission than any single measure used in isolation [17]. This model was recently adapted to show the necessity of scaling the oviposition site encounter rate to the searching ability of the mosquito [20]. Le Menach et al. (2007) described an elaborate feeding cycle model that included an equivalent delay in the gonotrophic cycle period as a result of ITN use. Mosquitoes that are not killed by the ITNs would experience a reduced rate of human encounters and this rate is a function of bednet coverage and the presence of alternative blood meal hosts [16], [21]. In all of these models, the gonotrophic cycle period is calculated as the inverse of the mosquito bite rate which is generally acknowledged to be the key parameter to target in theoretical studies of malaria [22].

In the present study, we model both the separate and combined impact of larval habitat source reduction and ITNs on reducing malaria transmission. We focus on the primary vector of malaria in Africa, *Anopheles gambiae* Giles *sensu stricto*, which is almost exclusively anthropophilic [23] and highly endophilic and endophagic [24]. In addition to the classic assumptions of the mechanisms by which these two control strategies reduce the basic reproductive number of malaria, we determine their effect on the gonotrophic cycle period of the mosquito. While both control strategies have the potential to incur delays in the gonotrophic cycle period that will impede the bite rate, we show how recent mathematical assessments of the impact of larval habitat destruction on the bite rate and malaria transmission may be overly optimistic. We describe the necessity of re-treating/replenishing ITNs in order to maintain a high insecticidal level and to avoid any potentially harmful effects on the human communities. Finally, we discuss the potential of ITNs and larval habitat source reduction used in conjunction for a more integrated vector management.

## Results

### Mosquito resource availability and *R_0_*

**Figure 1:**
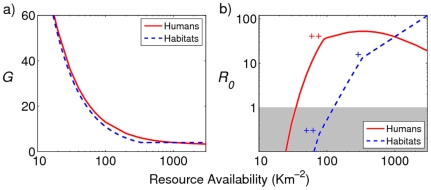
Resource availability influences the a) mosquito gonotrophic cycle, ‘*G*’, and b) *R_0_* of malaria. Mosquito resources consist of human blood meals and larval habitats. Availability of the unvaried resource was maintained at 1,000 per sq Km. “+” denotes the point at which the delay in locating oviposition sites equals the embryogenesis duration, and “++” denotes the point at which the gonotrophic cycle duration equals the extrinsic incubation period of malaria. The gray area highlights the region of *R_0_*<1, where malaria fails to persist. Results for mosquitoes with a searching ability of 1,000 sq m per day are shown.

*Anopheles gambiae* mosquitoes need human blood meals for egg production and larval habitats for oviposition. Therefore, human blood meals and larval habitats are considered important resources for the mosquitoes. Intuitively, as resources become more available, delays in the gonotrophic cycle (*G*) resulting from the finite searching ability of the mosquito (*s*) are reduced (all variables are defined along with their units in Table 1). Consequently, the bite rate increases, and with it, the basic reproductive number of malaria (*R_0_*). An interesting finding illustrated in Fig. 1a is that beyond a threshold, increased larval habitat density (*θ*) does not confer any additional reduction in the delay in the gonotrophic cycle. At this point, the duration of embryogenesis exceeds the delay in locating breeding sites 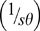. Hence, the duration of the cycle consists of the maturation time of the embryos (*ε*) summed with the delay in locating the blood meal (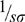, where *σ* is human host density).

**Table 1:** Definitions (and units) of the model variables and parameters.

Symbol	Definition (units)
*σ*	Human density (per Km^2^)
*θ*	Breeding site density (per Km^2^)
*m*	Ratio of mosquitoes to humans (mosquitoes per human)
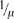	Average mosquito lifespan (days)
*P*	Mosquito daily survival
*T*	Extrinsic incubation period of malaria (days)
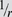	Average human infectious period (days)
*G*	Gonotrophic cycle period (days)
*ε*	Duration of embryogenesis (days)
*s*	Search ability of the mosquito (m^2^ per day)
*c*	Bed net coverage proportion
*ω*	Bed net killing efficacy

When larval habitats are abundant, habitat source reduction has a nearly linear effect on the basic reproductive number of malaria under our assumptions (Fig. 1b). Such an effect stems from proportional reduction in adult mosquito abundance as a consequence of habitat resource removal. However, when habitat availability is below the threshold density level (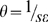), larval habitat resource reduction confers additional impact on *R_0_* due to its effect on the gonotrophic cycle duration. Consequently, reduction of *R_0_* is far more rapid when habitat source availability is lower. This makes biological sense; eliminating breeding sites has a proportionally greater impact when locating them becomes a rate-determining step in the mosquito's gonotrophic cycle.

For a given breeding site density, there is an optimum human density whereby the reduction in *R_0_* through a decreased mosquito to human ratio, *m*, is maximally countered by the increased *R_0_* through decreased *G* (the apex of the red curve in Fig. 1b). Hence, by ‘optimal human density’, we are referring to the density of humans that maximizes the *R_0_* of malaria. Below this optimum, an increase in human density is met with an increase in *R_0_*. Beyond this optimum, *R_0_* decreases with increasing human density as a result of a reduced *m* outweighing the effects of a shortened blood-seeking delay. The human density at which this point of inflection occurs, σ*, is dependent on the search capabilities of the mosquito, the embryo maturation time and the availability of breeding sites, and is calculated in the methods section (Equation 3).

### ITN coverage and *R_0_*

**Figure 2:**
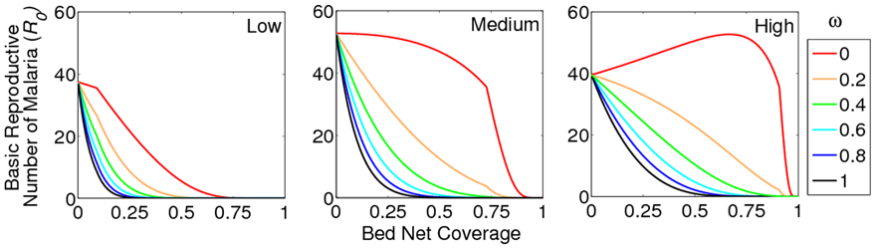
*R_0_* of malaria relative to the coverage and mosquito killing efficiency (*ω*) of ITNs. Results are shown for when human density is a) ‘Low’ (100 per sq Km), b) ‘Medium’ (333 per sq Km) and c) ‘High’ (1,000 per sq Km) and for mosquitoes that search 1,000 sq m per day. Larval habitat density is maintained at 1,000 per sq Km.

We examined the effects of ITNs on the *R_0_* of malaria under different coverage rates and mosquito killing efficacies (Fig. 2). The ITN coverage rate is defined as the proportion of human hosts sleeping under ITNs, assuming all residents are equally preferred by mosquitoes for blood meals. Figure 2 shows that even when human density is high, only around 50% of the community needs to be protected by 100% effective bed nets in order for the *R_0_* of malaria to fall below 1 (i.e. malaria fails to persist locally). Interrupting malaria transmission is shown to be more amenable for human populations of lower densities. Incomplete killing by the insecticides due to bed net deterioration (or resistance in the mosquito population) results in a requirement for greater bed net coverage. For example, when human density is high and the ITNs are only 40% effective, approximately 75% coverage is necessary for *R_0_* to be driven below 1 (Fig. 2). Other models of malaria have shown similar results [16], [25]. Of concern is when the insecticidal properties of bed nets are seriously compromised (when their mosquito-killing efficacy, *ω*, is in the order of ≤0.2) in human settlements of medium-to-high density. At this point and beyond, our analysis shows that the target of an 80% bed net coverage rate as proposed by the WHO's Roll Back Malaria program is insufficient to eliminate local malaria transmission. Of additional concern are studies such as Arredondo-Jiménez et al. (1997) which have found in the case of *Anopheles albimanus* that quite a high proportion of female mosquitoes (up to nearly 40%) could secure a bloodmeal from people sleeping under a bednet [26]. With a simple addition to our model (bednet coverage, *c*, is multiplied by a maximum protection threshold) we show in the supplementary material (Figure S1) that allowance for this factor has intuitive, but important, implications. Namely, that even 100% bednet coverage is not necessarily sufficient to prevent malaria's persistence and that incorporating this imperfection of bednet coverage necessitates a stricter threshold of mosquito-killing efficacy to drive *R_0_* below unity.

Figure 2 also illustrates how bed nets that have lost their insecticidal properties can actually exacerbate malaria transmission. While the ITN users will still be afforded protection from the physical barrier of the netting, the non-users will experience an increased bite rate as the blood seeking mosquitoes are simply deflected away from the non-lethal bed net users.

This potentially undesirable effect is explained by the relationship between *R_0_* and human density as illustrated in Figure 1b. Non-lethal bed nets affect *R_0_* on two fronts: they increase the number of mosquitoes taking blood meals from the non-user humans (increasing *R_0_*) but they also incur a delay in securing a blood meal (decreasing *R_0_*). Whether or not the overall effect is an increase or decrease in *R_0_* is dependent on the human density. Only at a low human density, will the increased bite rate experienced by the non-users of bed nets be negated by the increased delay in the mosquito seeking a blood meal. Hence, this undesirable effect is only experienced when the human population density exceeds the optimal level portrayed in Figure 1b and calculated in Equation 3.

**Figure 3:**
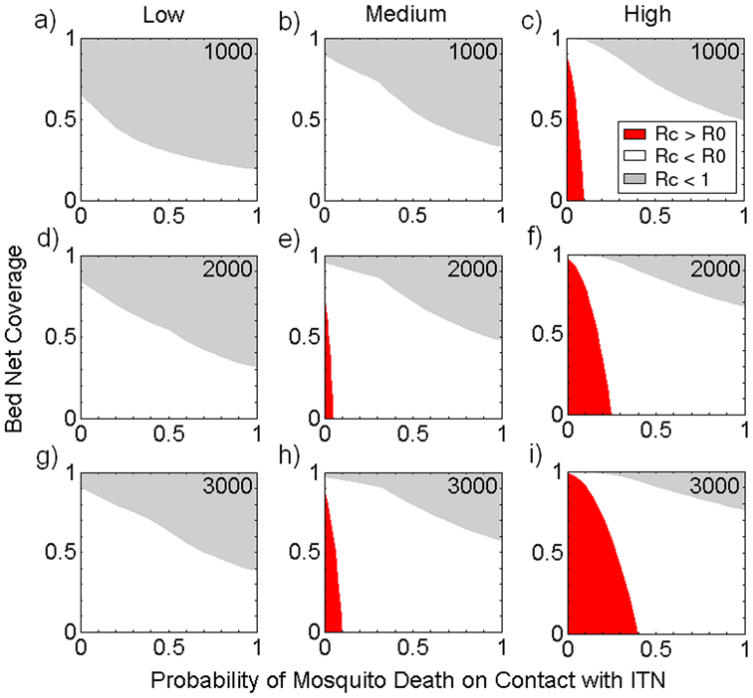
Influence of mosquito search ability on critical thresholds for the persistence of malaria. Letting *R_0_* and *R_c_* respectively denote the reproductive number in the absence and presence of control, the three parameter spaces are represented thusly: red (*R_c_*>*R_0_*, worse than no control), white (*R_c_*<*R_0_*, suppression but not elimination) and gray (*R_c_*<1, elimination of malaria transmission). Results are shown for a range of resource-locating abilities of the mosquito: 1,000 sq m per day (in a, b and c), 2,000 sq m per day (in d, e and f) and 3,000 sq m per day (in g, h and i). Human density values used in the simulation were: ‘Low’ (100 per sq Km in a, d and g), ‘Medium’ (333 per sq Km in b, e and h) and ‘High’ (1,000 per sq Km in c, f and i). Larval habitat density is maintained at 1,000 per sq Km.

Figure 3 illustrates how achieving the threshold necessary for local malaria elimination is compounded further by mosquitoes that are more adept at locating their resources. It also shows how potential for the inadvertent increase in *R_0_* occurs more readily as mosquitoes that are better searchers are simulated. For example, while an increased *R_0_* cannot occur in medium density human populations when the mosquitoes are poor searchers, it can occur when mosquitoes have increased search ability. This is because the optimal human density for disease persistence is reduced as a function of the increased mosquito search ability (see Equation 3).

### Integrating Habitat Resource Management with ITNs

**Figure 4:**
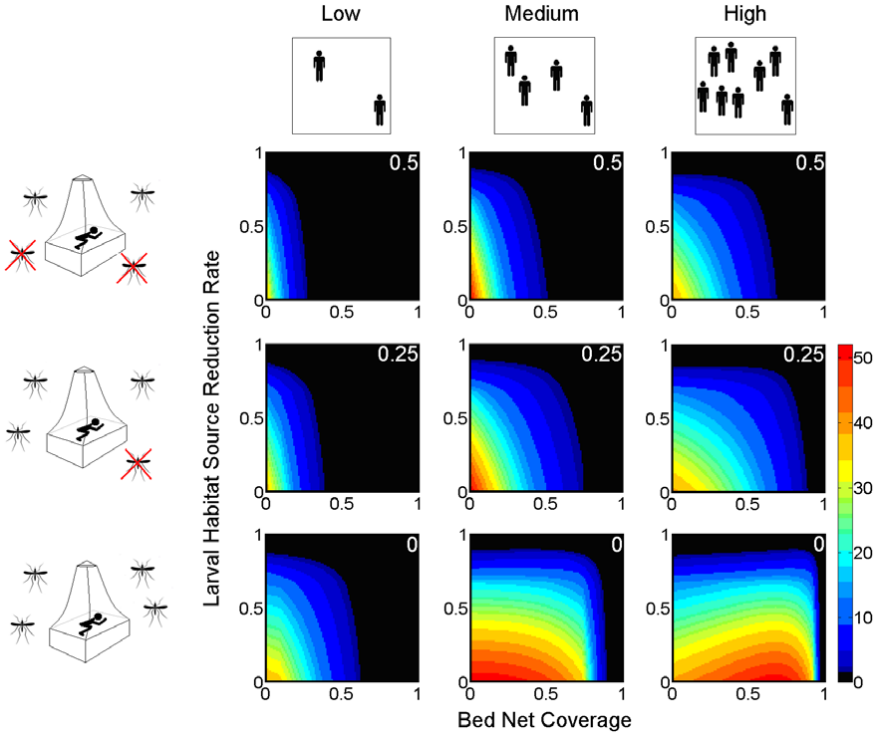
ITNs and habitat source reduction in the integrated control of malaria. The colors correspond to the basic reproductive number of malaria (*R_0_*) as highlighted in the key (the parameter space for local malaria elimination, *R_0_*<1, is black). The values in white font at the top-right corner of the plots indicate the killing efficacy of the ITNs: 0.5 (a, b and c); 0.25 (d, e and f); and 0.0 (g, h and i). Three human densities are used: ‘Low’ (100 per sq Km) (a, d and g), ‘Medium’ (333 per sq Km) (b, e and h), and ‘High’ (1,000 per sq Km) (c, f and i). Other parameters used in the simulation are mosquito search ability 1,000 sq m per day, and larval habitat density 1,000 per sq Km.

Figure 4 shows the effects of combining ITNs of varying mosquito-killing efficacies with larval habitat elimination for a range of human densities. Provided the bed nets are at least partially lethal to mosquitoes, *R_0_* is reduced more effectively by increasing bed net coverage than by attempting to complement ITNs with environmental management. This is illustrated by the more pronounced decrease in *R_0_* generally seen with increased ITN coverage (across the x-axis) than with increased habitat destruction (up the y-axis) in Figure 4. Integrating both control measures would seem more appropriate for locations of high human density, where the efficiency of the alternate strategies approaches equivalency. Combining both strategies becomes most beneficial when ITNs are non-lethal to mosquitoes and when human density is medium-to-high. Under such conditions, both a high rate of habitat destruction and a high ITN coverage are required to drive *R_0_* below 1.

**Figure 5:**
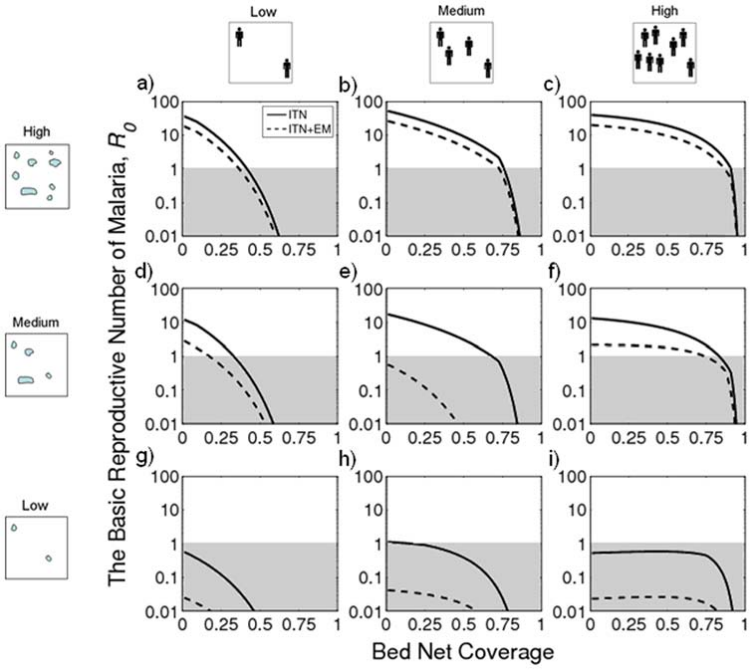
ITNs used alone or in conjunction with environmental management (EM) for malaria control. Parameters used in the simulation are as following: human density is ‘Low’ (100 per sq Km; a, d and g), ‘Medium’ (333 per sq Km; b, e and h), and ‘High’ (1000 per sq Km; c, f and i); larval habitat density is ‘Low’ (100 per sq Km; a, b and c), ‘Medium’ (333 per sq Km; d, e and f), and ‘High’ (1,000 per sq Km; g, h and i); mosquito search ability is 1,000 sq m per day; killing efficacy of the ITNs is 0.25; and habitat source reduction rate is 0.5.

Figure 5 also shows the relative merits of combining source reduction with ITNs for a range of human and breeding site densities. The figure shows how combining both control strategies is more effective in reducing *R_0_* when breeding sites are naturally at a density that is low enough to impose a delay in the mosquito's gonotrophic cycle. Very little benefit arises through integrating larval habitat resource reduction with ITNs when mosquito breeding sites are at a naturally high level. The greatest benefit of integrating these vector management tools is therefore when the insecticidal efficiency of bed nets is notably compromised and when the larval habitats are at a naturally low level.

## Discussion

Here, we described the conditions under which habitat source availability might be expected to significantly impact the risk of malaria epidemics. It has been suggested that larval habitat management acts multiplicatively in reducing *R_0_* by decreasing mosquito growth potential and extending the gonotrophic cycle duration [17], [20]. We have shown that habitat source availability and *R_0_* have a more complicated relationship than previously reported. We split the gonotrophic cycle into three components: the time required for mosquitoes to secure a blood meal, the embryo maturation duration and the delay in locating a larval habitat for oviposition. We show how a synergistic benefit in reducing *R_0_* through habitat source management is only applicable when larval habitats are so sparse as to delay oviposition for longer than the duration of embryogenesis. In other words, for oviposition to become a rate-determining step in the gonotrophic cycle, gravid *Anopheles gambiae s.s.* mosquitoes would have to take more than (in the order of) 3 days to seek out breeding sites.

We also examined the relationship between the gonotrophic cycle and the availability of human blood meals. While it is generally acknowledged that malaria control initiatives ought to be adapted to specific locations with regards to endemicity [27], seasonality [28], topography and land use [29], blood meal density rarely makes the cut. Many malaria-endemic or epidemic-prone regions in Sub-Saharan Africa, however, are experiencing rapid human population increases [30]. In examining the role of human density in the mosquito's gonotrophic cycle, we highlight its unusual relationship with the risk of malaria epidemics. Malaria persistence is human density dependent, and the optimum human density for malaria persistence is dependent on the duration of embryogenesis, the density of breeding sites and the search capabilities of the vector.

One mode of action of ITNs is that they reduce the availability of human hosts for blood feeding and the mosquito spends more time locating a blood meal as a consequence. Although it is generally acknowledged that bed nets therefore benefit the whole community and not just the users [5]–[7], some recent empirical evidence suggests otherwise [31]. Depending on whether human density is above or below the optimum human density (and the true resource-locating ability of the vector) we show how ITNs might actually increase the probability of malaria persistence by increasing the ratio of mosquitoes to available humans. This is only applicable, however, when the mosquito-killing efficacy of the ITNs is seriously compromised through insecticide resistance of the vector or through the depletion of the bed nets' insecticidal properties over time.

Concern for this detrimental effect was first voiced decades ago [32], [33], where heterogeneities in bite rates across a population were shown to increase *R_0_* relative to homogenous mixing. Recently, Smith et al. [34] suggested that when heterogeneous bite rates are accounted for, calculations of *R_0_* may underestimate by as much as ten-fold. Again, the search capability of *An. gambiae*–an aspect of vector ecology for which data is almost completely absent - has a marked impact on the likelihood of this occurrence. Quantifying the potential of this threat is therefore not possible until mosquito searching ability is better understood. Once details of this behavior are known, it might be prudent to allow for two separate variables in denoting ovipositional searching and host searching. The formulation of our model could easily allow for this future adaptation.

There is concern that ITN use will decrease in time as the community experiences initial alleviation from malaria morbidity [35]–[37]. Combining this shift in human behavior with the reduction in killing efficacy of the nets over time could result in conditions that are even more permissive of malaria transmission exacerbation through inadequate control. Longitudinal data on human bed net use fidelity as well as insecticidal half-life of long-lasting insecticidal nets are paramount to making realistic projections for their efficacy in combating malaria in the longer term.

We show interesting results for integrating habitat management with ITNs where, in general, only minor improvements in reducing *R_0_* resulted. It was hoped that as both measures might be expected to reduce the bite rate [16], [17], using both together would act synergistically. We did not find this to be the case. Firstly, the gonotrophic cycle is affected at two distinct stages by the strategies: ITNs that fail to kill the mosquito extend its host-seeking delay while a reduction in breeding sites affects ovipositional searching. Hence, at best, the effects are additive, not multiplicative. Second, breeding sites would have to be sufficiently scarce as to necessitate three days of ovipositional searching before this behavior becomes rate-determining in the gonotrophic cycle.

Our findings suggest that habitat source reduction would be of greatest use as a supplementary tool for malaria transmission reduction when it can be achieved to such a level that ovipositional searching becomes rate-determining in the gonotrophic cycle. Hence, environmental management is expected to be of greatest benefit when larval habitats are naturally scarce. Additionally, combining both strategies is more effective when human populations are higher and when the killing-efficacy of the bed nets is significantly compromised.

As with other theoretical studies, in order to present transparent relationships between outcomes and causes we have had to simplify the system significantly. There are numerous aspects that we feel warrant further analysis. The gonotrophic cycle duration is not only affected by resource density, but by multiple biotic and abiotic factors. Combining the effects of the local microclimate [38] and land use [39] will generate a determinant of the gonotrophic duration that can be expected to be highly variable over relatively small areas [40]. How the constituent behaviors involved in the gonotrophic cycle, and their associated mortality rates, are each affected under different environmental settings and control scenarios is a matter of keen interest [16], [21], [41], [42]. Also, whether our conclusions can be extended to scenarios in which larval habitats have large variation in mosquito productivity also warrants further analysis. It might be expected, for example, that focusing environmental management on more productive larval habitats would yield a higher level of efficacy [13], [43], although, this notion is contentious [44]. How the threshold resource densities calculated in this analysis are likely to vary within and between communities as a function of these and other additional complexities requires further development.

## Methods

We modeled the effects of larval habitat source reduction and the use of ITNs on the basic reproductive rate of malaria, *R_0_*. With a minor modification to the model of Macdonald (1957), *R_0_* is expressed as:



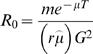
(1)


Here, *m* is the ratio of mosquitoes to people, *μ* is the force of mosquito mortality [45], *T* is the extrinsic incubation period of malaria, *r* is the recovery rate and *G* is the average length of the gonotrophic cycle. *μ* can be estimated by −ln(*P*), where *P* is the daily mosquito survival rate [45]. In the event of 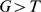, *G* replaces *T* in the exponential term of the nominator i.e. the female mosquito must survive the gonotrophic cycle period as well as the extrinsic incubation period of malaria before it can make a secondary, infectious bite. All parameters used in the present study are defined in Table 1.

### Larval Habitat Source Reduction and *R_0_*

Larval habitat source reduction decreases *R_0_* through both the ratio of mosquitoes to humans (*m*) and the gonotropic cycle duration (*G*) variables. If we assume that 1) larval habitat distribution is random, 2) all habitats are equivalently suitable, 3) gravid female mosquitoes randomly select larval habitats for oviposition, and 4) the relationship between larval habitat abundance and adult mosquito abundance is linear, we expect the larval habitat source reduction will reduce *m* proportionally with the rate of habitat source reduction, and the gonotrophic cycle duration will increase as the gravid mosquito takes longer to locate oviposition sites. Assuming that females seek out potential oviposition sites while the embryos develop, this searching behavior only becomes rate-determining in the gonotrophic cycle if it exceeds the delay incurred by embryogenesis. Therefore, the average length of the gonotrophic cycle (*G*) is calculated as:



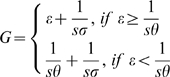
(2)


Where *ε* is mosquito embryo development duration, *s* is mosquito searching ability, *σ* is human density, and *θ* is larval habitat density. This expression differs from previous studies [20] by allowing for a realistic minimum delay of embryogenesis and treating the delay in locating oviposition sites as independent from the delay incurred in locating a blood meal. In other words, the elimination of breeding sites can affect the delay in oviposition without affecting the delay in locating human hosts.

While reducing the number of breeding sites (through habitat destruction, for example) reduces *R_0_* through both the *m* and *G* terms, the human population density affects *R_0_* through two opposing mechanisms: *R_0_* is decreased for settlements of high human density through a decreased *m*, however, it is increased through the *G* term. Under the condition that *G*<*T*, the optimal human density for the persistence of malaria, σ*, can be calculated:



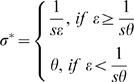
(3)


**(Details of this calculation can be found in Appendix S1).**

### Combining Larval Resource Management with ITNs

ITNs not only impose mosquito mortality, but also reduce the availability of blood meals to mosquitoes, increase host searching time, and subsequently prolong the mosquito gonotrophic cycle duration. Therefore, *R_0_* in the situation of simultaneous use of ITNs and larval habitat source reduction is expressed as:



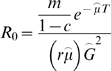
(4)


where *c* is the proportion of residents using bed nets, 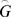 and 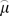 are the gonotrophic cycle duration and the force of mosquito mortality (respectively) once the effects of ITNs are factored in. In this formulation of the basic reproductive number of malaria, ITNs act on two additional fronts. First, the gonotrophic cycle duration is extended (becoming 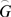) to cater for the fact that mosquitoes will be forced to seek hosts for longer:



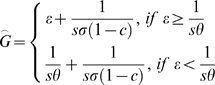
(5)


Second, the mosquito's force of mortality is then the composite effects of innate mosquito mortality and ITN-induced mortality among mosquitoes that come into contact with ITNs while seeking blood meals. If 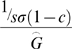 denotes the proportion of the gonotrophic cycle period that is involved in host-seeking, then mosquito mortality can be adjusted (becoming 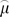) to allow for additional death caused by ITNs while seeking hosts:



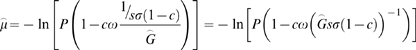
(6)


where *ω* is the mosquito killing efficacy of ITNs. Hence, as bed net coverage increases, so too does the delay in blood-seeking, 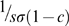, during which time the mosquito's force of mortality is increased. Equation 6 describes the additional impact of ITNs only on the mortality rate of mosquitoes that are actually host-seeking.

#### Parameters used in mathematical model

Parameters values which are typical of malaria-endemic settings in Africa were used. *An. gambiae* embryo development time (*ε*) is assumed to take 3 days [40]. The recovery rate (*r*) is the reciprocal of the average human infectious period (100 days). Mosquito daily survival rate (*P*) was estimated to be 0.85 [46]. The extrinsic incubation period of the malaria parasite (*T*) is assumed to be 14 days. Analysis was performed on the effects of larval resource management and ITNs both independently and in conjunction across a broad range of base-line human and breeding site densities (10 to 3,000 per Km^2^) and for mosquitoes of varying resource-searching abilities (1,000 to 3,000 m^2^ per day).

## Supporting Information

Appendix S1 The calculation of optimal human density
(0.05 MB DOC)

Figure S1 R0 of malaria relative to the coverage and mosquito killing efficiency (ω) of ITNs. In the top-right of the plots is the maximum extent by which mosquito-human contact is eliminated by bed net protection (1, 0.8 and 0.6). For example, ‘0.6’ indicates that sleeping under an ITN only reduces the person's chances of being bitten by 60%. Results are shown for a ‘High’ human population and larval habitat density (1,000 per sq Km) and for mosquitoes that search 1,000 sq m per day.
(0.05 MB TIF)
