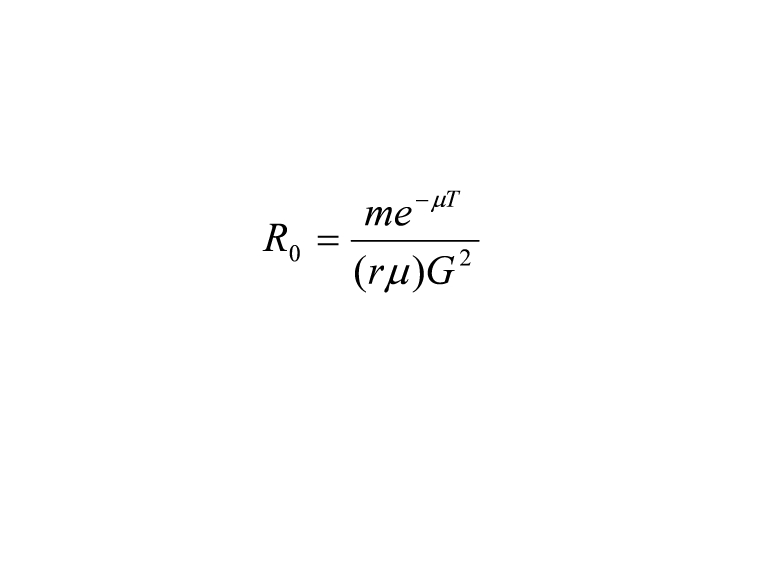# Correction: Modeling the Effects of Integrating Larval Habitat Source Reduction and Insecticide Treated Nets for Malaria Control

**DOI:** 10.1371/annotation/9d928eac-d6d9-4e75-bb1a-3a25c930c77f

**Published:** 2009-11-02

**Authors:** Laith Yakob, Guiyun Yan

There was an error in one of the symbols of Equation 1. Please view the correct equation here: