# Supplementary material for: Modeling the Effects of Integrating Larval Habitat Source Reduction and Insecticide Treated Nets for Malaria Control
Source: PLoS One. 2009 Sep 9;4(9):e6921. doi: 10.1371/journal.pone.0006921 (PMC2734167; doi:10.1371/journal.pone.0006921)
Supplement: Appendix S1 — The calculation of optimal human density (0.05 MB DOC) [file pone.0006921.s001.doc]

**Calculating the optimal human density, σ***

As stated, ‘optimal human density’ refers to the density of humans at which R_0_ is maximized. We therefore start with the equation for R_0_:

$R_{0}=\frac{me^{-\mu T}}{(r\mu)G^{2}}$ **Equation A1**

We separate the elements which are affected by human density from the elements which are not:

$R_{0}=\frac{m}{G^{2}}\times\frac{e^{-\mu T}}{r\mu}$ **Equation A2**

Hence:

$R_{0}\propto\frac{m}{G^{2}}$ **Equation A3**

Higher human density (*σ*) decreases *m* by a factor of 1/*σ*, therefore in order to maximize R0, we want to maximize the expression:

$\frac{1}{\sigma G^{2}}$ **Expression A4**

Beginning with the situation whereby $\varepsilon\geq\frac{1}{s\theta}$, we want to maximize:

$\frac{1}{\sigma\left(\varepsilon+\frac{1}{s\sigma}\right)^{2}}$ **Expression A5**

Multiplying the brackets out results in:

$\frac{1}{\sigma\varepsilon^{2}+\frac{2\varepsilon}{s}+\frac{1}{s^{2}\sigma}}$ **Expression A6**

Maximizing Expression 6 is the same as minimizing its inverse. To do this, we have to find the derivative of the inverse of Expression 6, set it to equal zero and solve for *σ*:

$f(\sigma)=\sigma\varepsilon^{2}+\frac{2\varepsilon}{s}+\frac{1}{s^{2}\sigma}$ **Equation A7**

$f'(\sigma)=\varepsilon^{2}-\frac{1}{s^{2}\sigma^{2}}$ **Equation A8**

$0=\varepsilon^{2}-\frac{1}{s^{2}\sigma^{2}}$ **Equation A9**

Which becomes:

$\sigma*=\frac{1}{s\varepsilon}$ **Equation A10**

Finally, substituting $\frac{1}{s\theta}$ for *ε* we can solve for the condition that $\frac{1}{s\theta}>\varepsilon$

$\sigma*=\frac{1}{s\frac{1}{s\theta}}=\frac{1}{\frac{1}{\theta}}=\theta$ **Equation A11**

Hence, we show that:

$\sigma*=\left\{\begin{matrix} \frac{1}{s\varepsilon}, & if & \varepsilon\geq\frac{1}{s\theta} \\ \theta, & if & \varepsilon<\frac{1}{s\theta} \end{matrix}\right.$ **Equation A12**
