# Supplementary figures and images for: Modeling the Effects of Integrating Larval Habitat Source Reduction and Insecticide Treated Nets for Malaria Control
Source: PLoS One. 2009 Sep 9;4(9):e6921. doi: 10.1371/journal.pone.0006921 (PMC2734167; doi:10.1371/journal.pone.0006921)

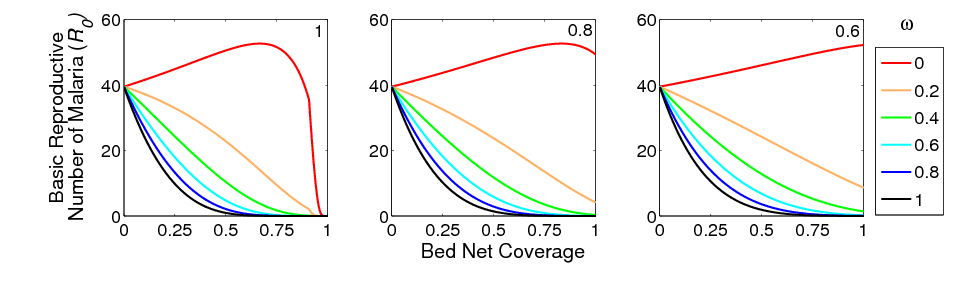

Supplement: Figure S1 — R0 of malaria relative to the coverage and mosquito killing efficiency (ω) of ITNs. In the top-right of the plots is the maximum extent by which mosquito-human contact is eliminated by bed net protection (1, 0.8 and 0.6). For example, ‘0.6’ indicates that sleeping under an ITN only reduces the person's chances of being bitten by 60%. Results are shown for a ‘High’ human population and larval habitat density (1,000 per sq Km) and for mosquitoes that search 1,000 sq m per day. (0.05 MB TIF) [file pone.0006921.s002.tif]
